# Neurosurgery and prognosis in patients with radiation-induced brain injury after nasopharyngeal carcinoma radiotherapy: a follow-up study

**DOI:** 10.1186/1748-717X-8-88

**Published:** 2013-04-11

**Authors:** Yi Li, Xiaolei Shi, Xiaoming Rong, Ying Peng, Yamei Tang

**Affiliations:** 1 Department of Neurology, Sun Yat-Sen Memorial Hospital, Sun Yat-Sen University, Guangzhou, China; 2 Key Laboratory of Malignant Tumor Gene Regulation and Target Therapy of Guangdong Higher Education Institutes, Sun Yat-sen University, Guangzhou, China

**Keywords:** Radiotherapy, Nasopharyngeal carcinoma, Radiation-induced brain injury, Neurosurgery

## Abstract

**Background:**

Radiotherapy is the standard radical treatment for nasopharyngeal carcinoma (NPC) and may cause radiation-induced brain injury (RI). Treatment for RI remains a challenge. We conducted this study to investigate the indications of neurosurgery, operation time and prognosis of patients with RI after NPC radiotherapy who underwent neurosurgical management.

**Methods:**

This was a follow-up study between January 2005 and July 2011. Fifteen NPC cases of RI who underwent neurosurgery were collected. Brain Magnetic resonance imaging (MRI), surgery and histology were studied. The outcome was assessed by LENT/SOMA scales and modified Rankin scale.

**Results:**

Brain lesion resection (86.7%) was more common than decompressive craniotomy (13.3%). According to LENT/SOMA scale before and six months after surgery, 13 of 15, 12 of 15, 14 of 15, and 14 of 15 cases showed improvement at subjective, objective, management and analytic domains, respectively. 12 of 15 patients showed improvement of modified Rankin scale after surgery. Three patients who underwent emergency surgery showed significant improvement (average score increment of 2, 2.7, 2.7, 3 and 2 at LENT/SOMA scale subjective, objective, management, analytic, and modified Rankin scale, respectively), as compared with 12 cases underwent elective surgery (average score increment of 1, 1, 1.4, 1.8 and 1 at LENT SOMA scale subjective, objective, management, analytic, and modified Rankin scale, respectively).

**Conclusions:**

Neurosurgery, including brain necrotic tissue resection and decompressive craniotomy, improves the prognosis for RI patients, especially for those with indications of emergency surgery.

## Introduction

Nasopharyngeal carcinoma (NPC) is the third most common malignant tumor in men in certain regions of East Asia, with an incidence of 15 to 50 per 100,000 [1]. Radiotherapy is the standard radical treatment for NPC and has produced long-term effects. However, it injuries the normal brain tissue and accounts for several severe delayed sequelae, including radiation-induced brain injury (RI) [2]. At the time patients present with clinical symptoms of RI, the brains have already suffered with irreversible damage.

Treatment for RI remains a challenge. Although rehabilitation and medication, e.g. corticosteroids, dehydrants, anticoagulants and neuroprotective agents, were the mainstay of treatment [3,4], some cases of RI showed exacerbation. Cerebral lesions in these cases might show as mass effect, necrosis, foci of calcification, encephalomalacia, and/or porencephaly [5,6], and these changes were reserved as signs of neurosurgical management [7]. In this study, we prospectively analyzed the indications, operation time and prognosis of RI patients after NPC radiotherapy who underwent neurosurgical management.

## Materials and methods

Adult NPC patients with radiation-induced brain injury after radiotherapy who underwent neurosurgery between January 2005 and July 2011 were prospectively analyzed. At least six months follow-up was performed.

### Patients

All patients were inpatients at Sun Yat-Sen Memorial Hospital in Guangzhou, China. Inclusion criteria were defined as follow: (1) a history of radiotherapy after NPC; (2) a diagnosis of RI based on magnetic resonance imaging (MRI), with or without neurological deficits; and (3) an exclusion of cancer relapse, metastases, other malignances, neurovascular disease, demyelinating disease, or other diseases of the nervous system. The operation indication of the patients was evaluated by two neurological surgeons and two neurologists. The institutional ethics review board of Sun Yat-Sen Memorial Hospital granted approval for the study (No. 审伦备[1]号). Informed consents were obtained from all involved subjects.

The following data were recorded:

Demographics: Age, sex, post-radiotherapy intervals (interval between radiation and diagnosis of RI, and interval between diagnosis of RI and neurosurgery), radiation dose, radiation field, and reirradiation.

Clinical examination: Patients were clinically assessed by two qualified neurologist, and underwent general and neurological examinations. Neurological symptoms were recorded.

Imaging: Brain MRI was diagnosed by a radiologist and a neurologist, and results were recorded.

Surgery: The standard type of surgeries was eradication of necrotic brain tissue or cyst. Decompressive craniotomy was used to relieve the elevated intracranial pressure.

Histology: Pathological results for hematoxylin and eosin staining after surgery were recorded.

Outcome of surgery: The outcome at the end of six months after surgery was assessed according to LENT/SOMA scales (late effects of normal tissue- subjective, objective, management, analytic) and modified Rankin scale.

### Statistical analysis

Multiple logistic regression was used to determine the association between age, gender, post-radiotherapy intervals, radiation dose, radiation field, brain MRI, surgery, pathology and clinical outcome. Testing was done at the 0.05 level of significance.

## Results

### Individual characteristics

The demographic data were summarized in Table 1. There were 15 patients with RI who were performed surgery, including 13 (86.7%) males and 2 (13.3%) females. The mean age was 50 ± 9.0 years, with a range of 28 to 65 years. The median interval between radiation and diagnosis of RI was 4 years, with a range of 0.5 to 13 years. The median interval between diagnosis of RI and surgery was 12 months, with a range of 3 to 144 months. The mean radiation dose was 83 ± 28 grays (Gy), with a range of 66 to 138 Gy. Radiation field included faciocervical portal or preauricular portal, with a radio of 11:4. Three of 15 patients underwent reirradiation.

**Table 1:** Demographic Data of RI patients underwent surgery (N = 15, in years)

**Case**	**Age**	**Gender**	**Interval 1(year)**^**1**^	**Interval 2 (month)**^**2**^	**Radiation dose (Gy)**	**Radiation field**	**Reirradiation**	**Follow-up months**	**Clinical outcome**
1	53y	M	0.5	144	68	Faciocervical	N	52	Survival
2	57y	M	5	36	76	Faciocervical	N	39	Survival
3	57y	M	7	36	70	Preauricular	N	32	Survival
4	60y	M	2	3	70	Faciocervical	N	25	Died
5	28y	M	3	10	68	Preauricular	N	30	Survival
6	58y	F	13	12	66	Faciocervical	N	8	Died
7	48y	M	3	5	70 + 68	Faciocervical	Y	6	Survival
8	57y	F	5	12	72	Faciocervical	N	60	Survival
9	42y	M	4	11	68 + 68	Faciocervical	Y	11	Survival
10	53y	M	4	11	68 + 68	Faciocervical	Y	13	Survival
11	42y	M	2	24	70	Preauricular	N	41	Survival
12	65y	M	7	12	70	Preauricular	N	27	Survival
13	51y	M	3	36	68	Faciocervical	N	71	Survival
14	48y	M	2	24	70	Faciocervical	N	20	Survival
15	50y	M	5	22	74	Faciocervical	N	22	Survival

^1^ Interval between radiation and diagnosis of RI (for the patients who underwent reirradiation, this was the interval between reirradiation and diagnosis of RI).

^2^ Interval between diagnosis of RI and surgery.

### Brain MRI

We performed brain MRI on these 15 RI patients. Areas of RI showed as prolonged T2 relaxation time in multiple brain lobes (46.7%), bilateral temporal lobes (46.7%), unilateral temporal lobe (6.7%) and brain stem (13.3%). As shown in Table 2, nine of 15 (60.0%) patients underwent surgery showed cystic regions, and seven cases (46.7%) showed mass effect and/or shift of the midline in the brain.

**Table 2:** MRI characteristics in RI patients underwent surgery

**Brain MRI foci**		**Case number**
Multiple brain lobes		7
Temporal lobe	Bilateral	7
	Unilateral	1
Brain stem		2
Cyst		9
Mass effect and/or shift of the midline		7

### Surgery and histology

Brain lesion resection was taken in 86.7% patients, and decompressive craniotomy in 13.3% patients (Table 3). Three patients underwent emergency surgery due to significantly increased intracranial pressure, unconsciousness and signs of brain herniation. As shown in Table 4, the commonest pathology was brain necrosis (100%), followed by gliosis (76.9%) and small vascular proliferation (69.2%). Haemorrhage (46.2%), edema (23.1%), and the infiltration of inflammatory cells (7.7%) were also found in RI patients.

**Table 3:** Type of surgical procedure

**Surgery**	**Brain lesion resection**	**Decompressive craniotomy**	**Total**
Case number	13	2	15

**Table 4:** Pathology

**Case**	**Pathology**					
	**Brain necrosis**	**Gliosis**	**Small vascular proliferation**	**Haemorrhage**	**Edema**	**Infiltration of inflammatory cells**
1	+	-	-	+	-	-
2	+	+	+	+	-	-
3	+	+	-	+	-	-
4	+	+	+	-	-	-
5	+	+	+	-	+	+
6	+	+	-	-	-	-
7*	-	-	-	-	-	-
8	+	-	+	-	+	-
9	+	+	+	+	-	-
10*	-	-	-	-	-	-
11	+	+	+	-	-	-
12	+	+	+	+	-	-
13	+	-	+	-	+	-
14	+	+	+	+	-	-
15	+	+	-	-	-	-
Total	13	10	9	6	3	1

* These two cases were performed decompressive craniotomy and pathological results were not displayed.

### Outcome

The outcome was analyzed in the 15 RI patients who underwent surgery.

The post-operative follow-up ranged from 6 to 71 months. Two patients died at the endpoint (Table 1). LENT/SOMA scales and modified Rankin scale were evaluated before surgery and six months after surgery. As shown in Table 5, most cases showed improvement at subjective, objective, management and analytic domains, respectively. 12 of 15 patients showed improvement of modified Rankin scale after surgery. Three patients who underwent emergency surgery showed greater improvement (average score increment of 2, 2.7, 2.7, 3 and 2 at LENT/SOMA scale subjective, objective, management, analytic domains, and modified Rankin scale, respectively), as compared with 12 cases underwent elective surgery (average score increment of 1, 1, 1.4, 1.8 and 1 at LENT/SOMA scale subjective, objective, management, analytic domains, and modified Rankin scale, respectively). No significance was shown in the statistical analysis of the association between age, gender, post-radiotherapy intervals, radiation dose, radiation field, brain MRI, surgery, pathology and clinical outcome (Table 6).

**Table 5:** Score increment of LENT/SOMA scales and modified Rankin scale before and after sugery

**Score Increment**		**+3**	**+2**	**+1**	**0**
LENT SOMA (case number)	Subjective	0	5	8	2
	Objective	2	3	7	3
	Management	3	6	5	1
	Analytic	5	7	2	1
Rankin (case number)		0	5	7	3

**Table 6:** Multiple logistic regression analysis of age, gender, post-RT intervals, radiation dose, radiation field, brain MRI, surgery, pathology to predict clinical outcome

**Variables**		**Score**	**Degree of freedom**	***P*value**
Age		1.799	1	0.180
Gender		2.685	1	0.101
Interval 1(year) ^1^		2.671	1	0.102
Interval 2 (month) ^2^		1.057	1	0.304
Radiation dose (Gy)		0.774	1	0.379
Radiation field		0.839	1	0.360
Surgery		0.355	1	0.551
Brain MRI foci	Multiple brain lobes	2.637	1	0.104
	Bilateral temporal lobes	2.019	1	0.155
	Unilateral temporal lobe	0.165	1	0.685
	Brain stem	0.355	1	0.551
	Cyst	3.462	1	0.063
	Mass effect and/or shift of the midline	2.637	1	0.104
Pathology	Brain necrosis	0.709	1	0.400
	Gliosis	0.410	1	0.522
	Small vascular proliferation	2.026	1	0.155
	Haemorrhage	0.709	1	0.400
	Edema	0.197	1	0.657
	Infiltration of inflammatory cells	5.318	1	0.378

Dependent: clinical outcome (survival or died).

^1^ Interval between radiation and diagnosis of RI (for the patients who underwent reirradiation, this was the interval between reirradiation and diagnosis of RI).

^2^ Interval between diagnosis of RI and surgery.

## Discussion

We have prospectively evaluated the therapeutic effectiveness of neurosurgery on radiation-induced brain injury. With respect to the scales evaluation before and after surgery, more than 80% patients achieved remission. Thus, our study indicated that neurosurgery intervention was beneficial as an invasive treatment. On the other hand, approximately 20% of our cases performed no improvement after surgery. The latter observation was likely due to unremitting cognitive impairment. No significance was shown in the statistical analysis of the association between individual characteristics, brain MRI, surgery, pathology and clinical outcome. This might be limited by the small sample size.

The patients who experienced recurrence of NPC would be retreated with a second course of radiotherapy. Whole brain reirradiation was reported to cause acute adverse reactions in more than 70% of patients [8]. Three cases of our patients suffered with NPC recurrence, and were administered with a second course of radiation. Severe brain injuries, shown as massive foci in multiple lobes of the brain and transtentorial herniation, occurred in these three patients shortly after reirradiation. One case was diagnosed with RI three months after reirradiation, and underwent surgery five months after diagnosis of RI, the other two showed severe headache, disturbance of consciousness, and obvious foci in brain MRI within one month after reirradiation, and underwent surgery about one year later. Mou et al. reported 14 cases of RI who underwent neurosurgery treatment, and found that the mean latency of radiation-induced brain necrosis in patients with reirradiation was much shorter than that with one course radiation [9]. Radiation-induced normal brain tissue necrosis is found to commonly occur at the cumulative normalized total dose of >100 Gy [10]. Thus, reirradiation increased the incidences of normal brain necrosis and edema, and might increase the probability of emergency surgery other than elective surgery. Moreover, clinical and scoring evaluation showed significant improvement after emergency surgery.

For patients with RI after NPC radiotherapy, surgery is considered to be the last resort for those who with poor-controlled conditions, e.g. progressive symptoms despite conservative therapy, hemorrhage, and brain necrosis formation [7,11]. In this study, the emergency surgery was performed in three patients who showed significantly increased intracranial pressure, unconsciousness, signs of brain herniation at admission, and the critical symptoms could not be alleviated quickly by conservative methods. With regard to elective surgery, indications and selections of operation time should be evaluated with more caution. Twelve of our 15 cases underwent elective surgery. The indications for elective neurosurgery included severe brain edema and necrosis (8 of 15), severe symptoms e.g. headache, disturbance of consciousness (5 of 15), and hemorrhage (1 of 15), and full-course medication, including intravenous corticosteroids, could not relieved these symptoms. There was one case in our study performed neurosurgery due to a misdiagnosis. The initial diagnosis based on imaging was metastasis of NPC. To be certain, differentiating this condition from brain tumor remains a challenge [12]. The differential diagnoses of RI include intracranial extension of NPC, second primary intracranial neoplasm, cerebral metastasis, meningeal spread and brain abscess [13]. Presently, MRI scan is commonly used for diagnosis of RI, and was sensitive in revealing radiation damage of the brain [14]. However, a definitive diagnosis cannot be obtained from MRI, or other imaging tools, e.g. CT and PET-CT. In brain imaging, both malignant lesions and radiation-induced injuries showed as marked vasogenic edema, mass effect, and ring-enhancing patterns [15,16]. Advanced imaging methods and computer technology, including diffusion-weighted magnetic resonance imaging, perfusion magnetic resonance imaging, and proton magnetic resonance spectroscopic imaging, would help to narrow the differential possibilities [7,17,18]. However, a definitive diagnosis of cancer still needed morphology. Furthermore, the MRI of our misdiagnosed patient presented brain lesion in the occipital and frontal lobes instead of the temporal lobe. This could be another cause of diagnostic dilemma.

## Conclusions

In summary, we observed the MRI imaging, surgical treatment, histology and prognosis in RI patients after radiotherapy of NPC who underwent neurosurgery. Our observation found that neurosurgery, including brain necrotic tissue resection and decompressive craniotomy, improved the prognosis for RI patients, especially for those with indications of emergency surgery.

## Abbreviations

NPC: Nasopharyngeal carcinoma; RI: Radiation-induced brain injury; MRI: Magnetic resonance imaging

## Competing interests

The authors have no conflict of interest.

## Authors’ contributions

YT designed this study, carried out the collection and the follow-up of all the patients, and drafted the manuscript. YL and XS carried out the collection and the follow-up of all the patients, performed the evaluation of scales and the statistical analysis, and drafted the manuscript. SR participated in the follow-up of all the patients, helped to perform the evaluation of scales. YP participated in the design of the study and coordination and helped to draft the manuscript. All authors read and approved the final manuscript.
